# Nearly one-in-five households utilized inadequate iodized salt in Nifas Silk Sub-City, Addis Ababa, Ethiopia

**DOI:** 10.1186/s40795-023-00754-5

**Published:** 2023-08-07

**Authors:** Getachew Sale Mezgebu, Endalkachew Amare Enyew, Beakal Zinab Tefera, Fentaw Wassie Feleke

**Affiliations:** 1 Department of Nutrition and Dietetics, Faculty of Public Health, Institute of Health, Jimma University, Jimma, Ethiopia; 2 Department of Human Nutrition, School of human nutrition and food science technology, College of agriculture, Hawassa University, P.O.BOX 05, Hawassa, Ethiopia; 3 Department of Public Health, School of Public Health, College of Medicine and Health Sciences, Woldia University, P.O.BOX 400, Woldia, Ethiopia

**Keywords:** Addis Ababa, Ethiopia, Iodine deficiency disorder, Iodized salt utilization

## Abstract

**Background:**

There is no country in the developing world where iodine deficiency is not a public health problem including Ethiopia. Therefore, this study aimed to assess inadequate utilization of iodized salt and associated factors at household level in woreda 11 Nifas Silk Sub-city, Addis Ababa, Ethiopia.

**Methods:**

A community-based cross-sectional study was conducted with multistage sampling technique on 348 household respondents. The data were collected using interviewer-administered structured questionnaires and an iodine rapid test kit. The data were edited, cleaned, and entered using Epi-data version 4.6.2 and exported to SPSS version 25 for analysis. A multivariable logistic regression model was fitted to identify associated factors for inadequate utilization of iodized salt. The statistical significance was declared at a p-value of less than 0.05 with 95% confidence interval.

**Results:**

A total of 348 household respondents were participated. The amount of iodine content in salt 0 ppm, < 15ppm and > 15ppm were 11.8%, 7.2% and 81.0% respectively. Total inadequate utilization of iodized salt was 19%. Using unpacked salt [AOR; 0.50 (95%CI: 0.27, 0.93)], using a container without a lid [AOR; 0.29 (95%CI: 013, 0.63)], and having insufficient knowledge [AOR; 2.10 (95%CI: 1.14, 3.86)] were all significantly connected with using inadequate iodized salt.

**Conclusions:**

Iodized salt utilization was inadequate. Using containers without a lid, unpacked salt, and poor knowledge were associated factors. There should be a provision of adequate knowledge about iodized salt, a proper storage and handling.

**Supplementary Information:**

The online version contains supplementary material available at 10.1186/s40795-023-00754-5.

## Introduction

Iodine deficiency disorders (IDD) affect more than 50 nations and are a serious public health issue. WHO estimates that there are around 2 billion people in the world [1]. Worldwide 30% [2] of the world population suffer from insufficient iodine intake below 100 µg/L [3]. Iodine deficiency is a public health problem throughout the world [4, 5]. In Europe (57%), the Eastern Mediterranean (54%), Africa (43%), Southeast Asia (40%), the Western Pacific (24%), and the Americas (10%) are the countries most affected [6]. In Africa, about 260 million people have inadequate iodine intake resulting in iodine deficiency states, which may be related to a 10–15% lowering of average intellectual capacity [7, 8].

Mental retardation, growth retardation, reproductive failure, high childhood mortality, impairments in nervous system development, goiter, physical slowness, and economic stagnation are all connected with IDD [9]. Iodine deficiency can reduce average intellectual quotient (IQ) scores by 13.5 points [10] and a mild iodine deficiency can cause a significant loss of learning ability [11]. In Ethiopia, one out of every 1000 is a cretin and about 50,000 prenatal deaths are occurring annually due to iodine deficiency disorders [12], 26% of the total population have goiter and 62% of the population is at risk of IDD according to the national survey made by the previous Ethiopian Nutrition Institute [13].

Iodine can be found in seafood, dairy products, iodine-rich soils, and minor amounts in the majority of other foods. Topsoil contains iodine naturally, but it has been damaged by deforestation, erosion, and flooding [14]. Iodine shortage in the diet results from this absence of iodine in food crops. Consequently, people need extra sources to consume the required levels [15]. Despite this, the WHO supported the Universal Salt Iodization (USI) programme, a highly cost-effective public health policy [16], and salt iodization campaigns were started in about 120 different nations worldwide. Thanks to USI [17], iodine deficiency diseases have been wiped out in 34 of these countries.

Despite the fact that worldwide iodine nutrition has vastly improved, 20 to 30% of pregnancies and hence babies continue to be disadvantaged by the usage of iodized salt [18]. Iodized salt coverage varies by region, ranging from 90% in Asia and the Pacific to 40–60% in Sub-Saharan Africa [19]. Furthermore, use varies significantly between countries, ranging from 10 to 90%. Sudan, Mauritania, Guinea-Bissau, and the Gambia, for example, use less than 10% of iodized salt, whereas Burundi, Kenya, Nigeria, Tunisia, Uganda, and Zimbabwe have met the USI objective [20]. In Ethiopia, the use of adequate iodized salt increased from 15% to 2011 to 89% in 2016 [21, 22]. According to research conducted in Ethiopia, iodized salt usage ranges from 55.2% in Tigray [23] to 8.7% in the Lalo Asabi District in west Ethiopia [24–26].

The Ethiopian government revitalized and launched universal salt iodization activities, as well as strategies for the virtual elimination of IDD. The Ethiopian quality and standards authority has set the iodine level as potassium iodate at 60–80 parts per million (PPM), after allowing for iodine losses during storage and distribution, and salt fortification with iodine has been a long-term and effective preventive measure against IDD [27, 28]. However, still, only 26% of the households are using adequate iodized salt [29] and 10.8 − 36% of women aged 15–49 years have been affected by goiter [29, 30].

In fact, IDD continues to be a major public health concern for people of all ages [29, 31]. There is little data on the iodine amount of salt at the retail and consumer levels, and studies have found that households in Ethiopian regions frequently utilize iodized salt insufficiently. Although they did not show how knowledge and practice influence the use of salt with an acceptable iodine level in the research area, the parameters that predicted coverage, adequacy, and consumption were also investigated. In order to evaluate iodized salt intake and related factors among families in woreda 11 Nifas Silk Sub-City, Addis Abeba, Ethiopia, the current study was conducted.

## Methods and materials

### Study area

Woreda 11 Nifas Silk sub-city is where the study was carried out. Woreda 11 contained 8 settlements. The woreda record states that the current estimated total population was 65,512, with 30,398 men and 33,114 women living in 5845 homes.

### Study design and period

A community-based cross-sectional study was conducted from September 1 to October 1, 2020.

### Source population and study population

The source population consisted of all houses in Addis Ababa’s woreda 11 Nifas Silk Sub-city. The study population consisted of households from randomly selected villages in Addis Ababa’s woreda 11 Nifas Silk sub-city. The houses that provided the actual response during data collecting time were chosen by systematic sampling as the study unit. Respondents who were suffering from a serious illness at the time of the visit were omitted from the study.

### Sample size determination

The sample size was calculated using the Epi info software version 7.08 by using double population proportion formula considering the assumptions of 95% confidence interval, (Z_α/2_ = 1.96), 80% power and factors that had association with iodized salt utilization in respondents educational status among no formal education and formal education 21.3% and 35.7% respectively; a study done Arsi zone, South East Ethiopia [32]. Based on the assumption, the total sample size became 334. Since the total households of the district were 5845 (which was less than < 10,000), therefore reduction formula was used (nf = n/(1 + n/N)). Where nf was the desired sample size and after adding (10% non-response), the final minimum required sample size became 348 households.

### Sampling technique and procedure

To guarantee the representation of all residents in Addis Abeba’s Nifas Silk Sub-City, a multistage sampling the technique was adopted. The villages were regarded as clusters since their traits were thought to be uniform. Three out of eight villages, or a total of eight villages, were randomly selected by the lottery system to make up the sub-city. Then, using the sample size interval (K = 5), the total household was divided to select the households from each of the selected villages in the last stage of the process.

### Study variables

Inadequate iodized salt utilization was the outcome variable. Socio-demographic variables (age, sex, marital status, educational status, occupation, family size, monthly income), knowledge about iodized salt utilization, attitude about iodized salt utilization, availability, accessibility and taste of iodized salt, iodized salt utilization (type of salt, expose salt to sunlight, place of salt storage, type of container used to store, the time when did they add the salt while cooking) were the independent variables.

### Operational definitions

Adequately iodized salt was defined as a salt that was fortified with iodine and has an iodine concentration of greater than or equal to 15 PPM [24, 32, 33], and when the salt contains an iodine concentration less than 15 PPM was classified as inadequate iodized salt [24, 32–34].

#### Good knowledge

when the participant scores more than or equal to the average for knowledge question scores and knowledge scores less than the average (50%) was labeled as poor knowledge [35].

#### Favorable attitude

for positive statements, those who responded including strongly agree and agree in a Likert scale with five possible responses, and unfavorable for negative statements those who responded disagree, strongly disagree and uncertain. Marking the total attitude score out of a hundred, those with a score of greater than 50% were rated to have a favorable attitude and those with a score below 50% an unfavorable attitude [35].

### Data collection tools and processes

The data were collected using a structured questionnaire adapted from iodized salt program assessment tool [25] and from similar related kinds of literatures [23, 24, 35, 36]. The questionnaire was first prepared in English and translated to the local language Amharic and then back-translated to English to check for its consistency. The socio-demographic characteristics, knowledge, attitude, and practice of iodized salt questions were contextualized with the study area. The questionnaires contained both open and close-ended questions. The questionnaires included a section for observing the type of container used to store the salt and the place of salt storage. Iodine rapid test kit was used to assess the use of iodized salt at the household level by trained nurses and midwives using an interviewer based structured questionnaire and the iodine levels in sampled salt were measured by the rapid test kits.

To assess the iodine content of the salt at the household level, interviewers asked households to provide a teaspoon of salt used for cooking. The salt was tested for iodine by using the iodine rapid test kit (MBI Kits International). MBI KITS was an improved iodized salt field test kit for salt fortified with potassium iodide. The test kit was contained 2 test solution ampoules of 10 ml; 1 recheck solution ampoules of 10 ml, 1 color chart, and 1 white cup.

### Procedure to test iodine content of the salt

Primarily a small cup was filled with salt and then it was spread on a flat surface. Two drops of the test solution were added to the surface of the salt by piercing the white ampoule with a pin and then the ampoule was gently squeezed. Then after within one minute, the color of the salt was compared with the color chart and the iodine content was determined. When it had no color that appeared on the salt (after one minute), on a fresh sample five drops of the recheck solution in a red ampoule and then added two drops of test solution on the same spot was added. Finally, it was compared the color with the color chart and determined the iodine content.

### Data quality control

A pre-test of 10% of the minimum calculated sample size was performed, as well as data collecting and monitoring. The questionnaire was thoroughly reviewed before entering data. To determine the iodine content of salt at the home level, trained data collectors followed standard techniques. The data collectors and supervisors were trained for three days.

### Data processing and analysis

The acquired data were reviewed, cleaned, and input into Epi-data software version 4.6.2 before being exported to SPSS version 25.0 for analysis. Frequency tables, graphs, and summary statistics were used to present the descriptive analysis. Bivariable analysis was used to examine the relationship between outcome and independent factors. All variables with a p-value < 0.25 in the bivariable analysis were deemed candidate variables for the multivariable logistic regression model. A multivariable logistic regression model was used to discover factors influencing insufficient iodine salt utilisation. A p-value of less than 0.05 was considered statistically significant. The degrees of connection between outcome and independent variables were calculated using an adjusted odds ratio with a 95% confidence interval.

## Results

### Socio-demographic characteristics

A total of 348(100% response rate) participants were interviewed in the study period. The mean (± SD) age of the respondents were 31.7(± 10.4) years and 100(28.7%) of the mother were in the age range of 18–24 years. Among the total respondents, 217(62.4%) were married and 136(39.1%) attended college and above. The majority of 285(81.9) were female and 171(49.1%) were private employees. Almost half 172(49.4%) of family sizes were 3–5. In the present study, almost 45% of respondents earn > 90 USD per month (Table 1).

**Table 1:** Socio-demographic characteristics among households in Woreda 11, Nifas silk Sub-city, Addis Ababa, Ethiopia, 2020

Variables		Frequency	Percent
**Age in years**	18–24	100	28.7
	25–29	78	22.4
	30–34	52	14.9
	35–39	43	12.4
	≥ 40	75	21.6
**Sex**	Male	63	18.1
	Female	285	81.9
**Marital status**	Married	217	62.4
	Single	112	32.2
	Divorced	12	3.4
	Widowed	7	2.0
**Educational status**	Unable to read and write	26	7.5
	Read and write only	18	5.2
	Primary school (Grade 1–8)	62	17.8
	Secondary school (Grade 9–10)	78	22.4
	Preparatory school (Grade 11–12)	28	8
	College and above	136	39.1
**Occupation**	Government employ	37	10.6
	Private employ	171	49.1
	Student	31	8.9
	House wife	109	31.3
**Family size**	Less than 3	126	36.2
	3–5	172	49.4
	Above 5	50	14.4
**Average monthly income per USD**	< 30	25	7.2
	30–60	111	31.9
	60–90	58	16.7
	> 90	154	44.3

### Participant’s knowledge about iodized salt

The majority 249(71.6%) of the respondent knew at least one effect of iodine deficiency and while 99(28.4%) knew of two or more effects of iodine deficiency. In the current study 322(92.5%) respondents had heard about iodized salt and the majority of mother 216(62.1%) knew that iodized salt prevents IDD. The majority of 147(42.2%) source of information is health professionals. In the present study 216(62.1%) participants had a favorable attitude toward iodized salt utilization (Table 2).

**Table 2:** Knowledge among households in Woreda 11, Nifas silk Sub-city, Addis Ababa, Ethiopia, 2020

Variables		Frequency	Percent
**Knows effects of iodine deficiency at least one**	Yes	249	71.6
	No	99	28.4
**Knows effects of iodine deficiency Two or more**	Yes	99	28.4
	No	249	71.6
**Heard about iodized salt**	Yes	322	92.5
	No	26	7.5
**Knows that iodized salt prevents IDD**	Yes	216	62.1
	No	132	37.9
**Source of information on utilizing iodized salt n (338)**	From social media	114	32.8
	Health professional	147	42.2
	Other	77	22.1
**Attitude status**	Unfavorable	132	37.9
	Favorable	216	62.1

### The practice of participants about iodized salt

The majority of 238(68.4%) participants used packed salt. Of some mothers 46(13.2%) were exposed salt to sunlight. Almost all of 333(95.7%) participants stored salt dry places. The majority of 314(90.2%) participants used containers with lids. More than half 193(55.5%) added salt after cooking (Table 3).

**Table 3:** Handling of iodized salt among households in Woreda 11, Nifas silk Sub-city, Addis Ababa, Ethiopia, 2020

Variables		Frequency	Percent
**Type of salt**	Packed	238	68.4
	Non-packed	110	31.6
**Expose salt to sun light**	Yes	46	13.2
	No	302	86.8
**Place of salt storage**	Dry place	333	95.7
	Moisture area	15	4.3
**Type of container**	Container with a lid	314	90.2
	Container without a lid	34	9.8
**Time of adding salt during cooking**	While cooking	155	44.5
	After cooking	193	55.5

### Availability, accessibility, and taste of iodized salt

Almost all 332(92.5%) respondents got iodized salt easily when needed. The price of iodized salt for the majority of 256(73.6%) of the respondents was affordable and the test of the iodized salt for the majority of 248(71.3%) respondents was no different from to the normal salt (Table 4).

**Table 4:** Availability, accessibility, and taste of iodized salt among households in Woreda 11, Nifas silk Sub-city, Addis Ababa, Ethiopia, 2020

Variable		Frequency	Percent
**Availability iodized salt when needed**	Yes	332	92.5
	No	26	7.5
**The price of iodized salt**	Expensive	92	26.4
	Affordable	256	73.6
**Iodized salt taste change**	Yes	98	28.2
	No	248	71.3

### The iodine content of household salt

**Fig. 1:**
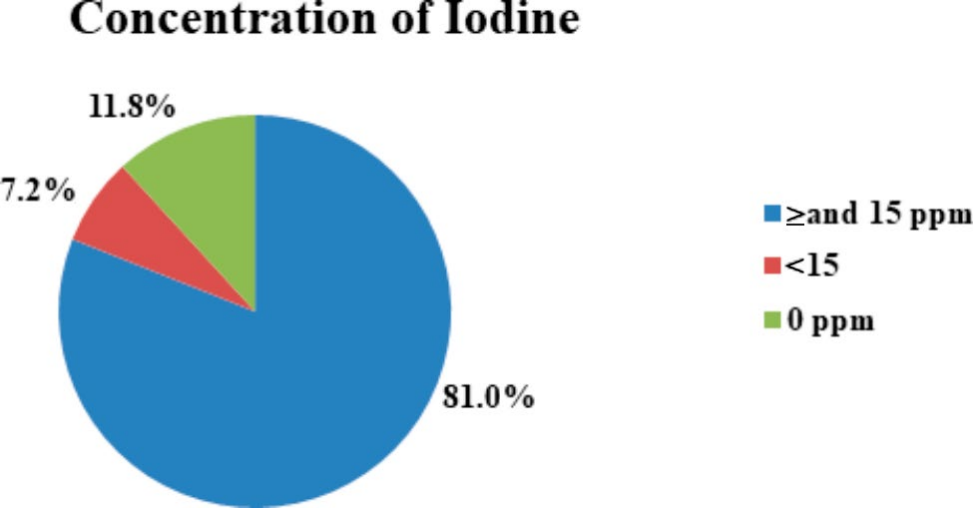
Percentage of iodine concentration in the salt among households in Woreda 11, Nifas silk Sub-city, Addis Ababa, Ethiopia, 2020

The prevalence of iodine content on salt 0 ppm, < 15ppm and ≥ 15ppm were 11.8%, 7.2% and 81.0% respectively (Fig. 1).

### Factors associated with inadequate utilization of iodized salt

Bivariable analysis was done to assess the association between individual independent variables and utilization of inadequately iodized salts. Age, family size, type of salt, type of container, time of adding salt during cooking, knowledge status, and attitude status became candidate variables for multivariable logistic regression model.

Finally keeping the other variables constant, the odds of utilization of inadequately iodized salt among the packed types of salt were 50% [AOR; 0.50 (95% CI= (0.27, 0.93)] lower than non-packed type users. The odds of inadequately iodized salt in a salt container with a lid were 71% [AOR; 0.29 (95%CI= (0.13, 0.63)] lower than a container with no lid. The odds of the utilization of inadequately iodized salt among respondents with poor knowledge were 2.29 times [AOR; 2.1 (95% CI = 1.14, 3.86] higher than respondents with good knowledge (Table 5).

**Table 5:** Bivariable and multivariable logistic regression model predicting factors associated with inadequate iodine salt utilization among households in Woreda 11, Nifas silk Sub-city, Addis Ababa, Ethiopia 2020

Variable		PPM		COR (95% CI)	P-value	AOR (95% CI)	P-value
		< 15ppm No (%)	≥ 15ppm No (%)				
**Age**	18–24	22(33.3)	78(27.7)	1.23(0.58,2.59)	0.590	1.63(0.73,3.62)	0.231
	25–29	19(28.8)	59(20.9)	1.41(0.65,3.05)	0.393	1.57(0.68,3.62)	0.293
	30–34	8(12.1)	44(15.6)	0.79(0.31,205)	0.631	0.68(0.25,1.88)	0.455
	35–39	3(4.5)	40(14.2)	0.33(0.09,1.21)	0.094	0.28(0.07,1.77)	0.064
	≥ 40	14(21.2)	61(21.6)	1		1	
**Family size**	< 3	30(45.5)	96(34)	2.29(0.89,5.91)	0.086	1.93(0.71,5.29)	0.201
	3–5	30(45.5)	142(50.3)	1.55(0.61,3.96)	0.361	1.49(0.55,4.06)	0.434
	> 5	6(9.1)	44(15.6)	1		1	
**Type of salt**	Packed	34(51.5)	204(72.3)	0.41(0.23,0.70)	0.001	0.50(0.27,0.93)	0.028*
	Not packed	32(48.5)	78(27.7)	1		1	
**Type of container**	Container with a lid	50(75.8)	264(93.6)	0.21(0.10,0.41)	0.001	0.29(0.13,0.63)	0.002*
	Container without a lid	16(24.2)	18(6.4)	1		1	
**Time of adding salt during cooking**	While cooking	34(51.5)	121(42.9)	1.41(0.83,2.42)	0.207	1.35(0.75,2.43)	0.313
	After cooking	32(48.5)	161(57.1)	1		1	
**Knowledge status**	Poor	40(60.6)	110(39)	2.41(1.39,4.16)	0.002	2.29(1.26,4.17)	0.007*
	Good	26(39.4)	172(61)			1	
**Attitude status**	Unfavorable	33(50)	99(35.1)	1.85(1.08,3.18)	0.026	1.28(0.69,2.40)	0.434
	Favorable	33(50)	183(64.8)			1	

*Significant at P-value < 0.05, AOR = adjusted odds ratio, COR: Crude Odds Ratio

## Discussion

The prevalence of iodine content on salt 0 ppm, < 15ppm and ≥ 15ppm were 11.8%, 7.2% and 81.0% respectively. In this study prevalence of inadequately iodized salt utilization was higher when compared to a study in Arsi at 8.2% [32]. This study was lower when compared to Tigray northern and western Ethiopia 44.6-82.5% [23, 37–39], Gambela 47.2% [40], Lalo Assabi district in West Ethiopia 92.3% [24], Jimma 73.8% [25], and Amhara regions 85% [26]. This might be due to the fact that a study area Addis Ababa was better socioeconomic status and also this might be due to the availability & accessibility of iodized salt in the market, legislation & policies to fortify salt with iodine, regular follow up & monitoring regarding utilization of iodized salt in this study area.

The current finding revealed that about 56.9% of participants had good knowledge about iodized salt & iodine deficiency disorders. The result was slightly higher when compared to a study done in Gobe town revealed about 51.7% of the respondents had sufficient knowledge about iodized salt and IDD [41]. It was also slightly higher than 56% in Sudan [42]. This finding was also lower when compared to a study done in Dire Dawa indicated that 62.4% of the households had good knowledge of the importance of using iodized salt [43]. It was also lower than in Yeka sub city Addis Ababa 78% [44]. This might be due to the different levels of social structure, regular follow up and monitoring regarding the utilization of iodized salt.

In this study about 62.1% of the respondents had a favorable attitude towards iodized salt which is higher than the study revealed from in Shebelle town 50.6% of respondents had a favorable attitude [35].

The study revealed that the odds of utilization of inadequately iodized salts among the packed types of salt were 50% lower than non-packed type users. This finding was also consistent with other studies like a study done in the Arsi zone, South East Ethiopia [34], Lalo Assabi District West Ethiopia [24], and Dire Dawa [43]. This might be due to the fact that exposing iodized salt to households or using unpacked salt gradually reduces iodine concentration in salt in addition to that of its volatile nature [45, 46]. However, iodine content will remain relatively constant if the salt is packed dry with an impermeable lining such as polyethylene bags [32] and the production might be contained inadequate iodine [47].

The odds of inadequate iodized salt in a salt container with a lid were 71% lower than a container with no lid. This study was also consistent with a study done in Lalo Assabi District, West Ethiopia [18], India [36], and Tigray [17]. This might be as iodine can be lost due to its volatile property [46].

The odds of the utilization of inadequately iodized salt among respondents with poor knowledge were 2.29 times higher than respondents with good knowledge. This finding was supported by another study done in Dire Dawa city and North west Ethiopia [39, 43, 46]. It was also supported by a study conducted in Bangladesh [48]. This might be due to the fact that unless households get access to information about how to store iodized salt, know the importance of it and the consequences of not using an ionized salt to reduce IDD. This was confirmed by the demographic analysis of findings from Ethiopian demographic and health survey [49]. The study limitation was on using only rapid field-testing kits to determine the availability of adequately iodized salt samples which did not include titration levels of iodine and urinary iodine.

## Conclusion

About 19% of households have inadequate iodine. Salt consumption, lack of knowledge, and unpackaged salt are all connected factors. Instructions and proper storage are also required. Future research can be conducted to assess the appropriateness of iodine concentrations at the manufacturing level.

### Electronic supplementary material

Below is the link to the electronic supplementary material.

Supplementary Material 1

## Data Availability

The data sets analysed during the current study are available from the corresponding author upon reasonable request.

## Abbreviations/acronyms

CSA: Central Statistical Agency
EDHS: Ethiopian Demographic & Health Survey
HH: Head of Household
ICCIDD: International Council for the Control of IDD
IDD: Iodine Deficiency Disorder
MOH: Ministry of Health
PPM: Parts Per Million
SPSS: Statistical Package for Social Sciences
TSH: Thyroid Stimulating Hormone
UNICEF: United Nations Children’s Fund
USI: Universal Salt Iodization
WHO: World Health Organization

## References

[1] Biban BG, Lichiardopol C (2017). Iodine Deficiency, still a global problem?. Curr Health Sci J.

[2] Hatch-McChesney A, Lieberman HR. Iodine and Iodine Deficiency: a Comprehensive Review of a re-emerging issue. Nutrients. 2022;14(17). 10.3390/nu14173474 PMC9459956 36079737

[3] Rami A, Saeid N, El Mzibri M, El Kari K, Idrissi M, Lahmam H (2022). Prevalence of iodine deficiency among moroccan women of reproductive age. Archives of Public Health.

[4] Ohlhorst SD, Slavin M, Bhide JM, Bugusu B (2012). Use of iodized salt in processed foods in select countries around the world and the role of food processors. Compr Rev Food Sci Food Saf.

[5] Andersson M, Takkouche B, Egli I, Allen HE, de Benoist B (2005). Current global iodine status and progress over the last decade towards the elimination of iodine deficiency. Bull World Health Organ.

[6] WHO. Iodine deficiency in Europe: a continuing public health problem. 2007.

[7] MI. Universal Salt Iodisation in Africa: road to overcoming the last hurdles. 2008.

[8] Bitamazire Businge C, Tafadzwa Musarurwa H, Longo–Mbenza B, Kengne AP. The prevalence of insufficient iodine intake in pregnancy in Africa: a systematic review and meta–analysis. 2019. 10.1186/s13643-022-02072-6 PMC9615360 36303220

[9] Venkatesh M. and John T. Salt Iodization for the Elimination of Iodine Deficiency. Amsterdam, Netherlands: 1st edition; 1995.

[10] Bhat S. Element of survival: isolating the causal effect of access to iodized salt on child health in India Harvard University, 2012 [updated 12 Feb 2021. Available from: http://thurj.org/research/2011/02/1628/.

[11] Luboshitzky R, Dgani YS, Atar S, Qupty G, Rakover Y. Tamir a thyroid dysfunction and goiter among immigrants from Ethiopia. PubMed 1994;127(9):289–93. 7843650

[12] Department FH, Ministry of health/United Nations Children’s Fund (1995). Nutrition policy: the miracle of iodated salt, Ethiopia’s commitment to salt iodation Joint Report on Situation Analysis.

[13] Assessment of the. iodine deficiency disorders and monitoring their elimination [Internet]. 2007.

[14] Duressa F, Mohammed Y, Feyissa R, Tufa T, Siraj K (2014). Comparative analysis of iodine concentration in water, soil, cereals and table salt of Horaboka, Mio and Besaso towns of Bale Robe, South East Ethiopia. J Environ Pollut Human Health.

[15] WHO, Iodine Status Worldwide, WHO Global Database on Iodine Deficiency. Geneva, Switzerland: World Health Organization Department of Nutrition for Health and Development; 2004.

[16] World Health Organization. Universal salt iodization and sodium intake reduction: compatible, cost-effective strategies of great public health benefit. 2022.

[17] Section UN, Division UP, UNICEF (2008). Communication UDo. Sustainable elimination of iodine deficiency: Progress since the 1990 World Summit.

[18] Maberly G, Haxton DP, Van der Haar (2003). Iodine deficiency: consequences and progress toward elimination. Food Nutr Bull.

[19] Mannar MV (2014). Making salt iodization truly universal by 2020. IDD Newsl.

[20] Jooste P, Andersson M, Assey V (2013). Iodine nutrition in Africa: an update for 2014. Sight Life.

[21] Central Statistical Agency and ICF International. Ethiopia Demographic and Health Survey. Addis Ababa, Ethiopia2011. p. 430.

[22] Centeral Statistical Agency and ICF International. Ethiopian Demographic Health Survey. Maryland, Calverton and Addis Ababa, Ethiopia2016.

[23] Desta AA, Kulkarni U, Worku KA, Sahle BW (2019). Iodine level concentration, coverage of adequately iodized salt consumption and factors affecting proper iodized salt utilization among households in North Ethiopia: a community based cross sectional study. BMC Nutr.

[24] Regassa M, Wolde T, Mulatu B. Utilization of adequately iodized salt on Prevention of Iodine Deficiency Disorders at Household Level and Associated factors in Lalo Assabi District, West Ethiopia. J Nutr Food Sci. 2016;6(2).

[25] Mezgebu Y, Mossie A, Rajesh P, Beyene G (2012). Prevalence and severity of iodine deficiency disorder among children 6–12 years of age in shebe senbo district, jimma zone, southwest Ethiopia. Ethiop J Health Sci.

[26] Zerfu D. National salt iodization coverage toward prevention of iodine deficiency disorder in Ethiopia. Addis Ababa, Ethiopia; 2014.

[27] Federal Democratic Republic of Ethiopia. National Nutrition Policy and Strategy. Addis Ababa, Ethiopia2004.

[28] FMOH. Ethiopa Federal Ministry of Health. Assessment of feasibility and potential benefits of food fortification in Ethiopia. Addis Ababa, Ethiopia; 2011.

[29] EPHI. Ethiopian Health and Nutrition Research Institute (2017). Goiter prevalence and median urinary iodine concentration in Ethiopian reproductive age women and school age children. Ethiop J Pub Health Nutr.

[30] International Council For Control of Iodine Deficiency Disorders. Senegal struggles to control iodine deficiency. 40. 2012.

[31] Abuye C, Berhane Y (2007). The goitre rate, its association with reproductive failure, and the knowledge of iodine deficiency disorders (IDD) among women in Ethiopia: cross-section community based study. BMC Public Health.

[32] Hiso DE, Roba KT (2019). Utilization of Iodized salt and Associated factor in Zuway Dugda District, Arsi Zone, Oromia Regional State, South East Ethiopia. East Afr J Sci.

[33] WHO. Assessment of Iodine deficiency Disorders andMonitoring their Elimination: A Guide for Programme Managers. 2001;2nd edition.

[34] Abebe Z, Tariku A, Gebeye E (2017). Availability of adequately iodized in Northwest Ethiopia: a cross-sectional study. Arch Public Health.

[35] Takele L, Belachew T, Bekele T. Iodine concentration in Salt at Household and Retail shop levels in Shebe Town, South West Ethiopia. East Afr Med J. 2003. 10.4314/eamj.v80i10.8757 15250627

[36] Tweneboa M. Determinants of the use of iodized salt among pregnant women attending Ante-natal clinic at the Kaneshie Polyclinic. 2015.

[37] Desta AA, Kulkarni U, Abraha K, Worku S, Sahle BW (2019). Iodine level concentration, coverage of adequately iodized salt consumption and factors affecting proper iodized salt utilization among households in North Ethiopia: a community based cross sectional study. BMC Nutr.

[38] Gemede HF, Tamiru B, Fite MB (2021). Knowledge, practice, and availability of Iodized Salt and Associated factors in Jibat Woreda, West Shoa Zone, Ethiopia. Int J Food Sci.

[39] Molla A, Giza M, Kebede F, Kebede T (2023). Iodine status, impact of knowledge, and practice for adequate iodized salt utilization in house hold at North West Ethiopia. SAGE Open Medicine.

[40] Tesfaye GA, Gemechu EN, Umer AY, Chanie FT. Prevalence of adequately iodized salt and its determinants in Gambela district, Southwest Ethiopia. medRxiv. 2023:2023.01. 17.23284676. 10.1136/bmjph-2023-000518 PMC11812733 40017856

[41] Tololu AK, Getahun FA, Abitew DB (2015). Coverage of Iodized Salt and Associated factors at Household Level in Goba Town, Bale Zone, South East Ethiopia. Sci J Public Health.

[42] Elmanssury A, Elnour SA, Elmosaad Y (2017). Knowledge and attitude of population towards iodized salt in Shendi locality river Nile state in Sudan. Eur Sci J.

[43] Ftwi G, Mengistie B, Abdo M, Roba KT (2018). Household Salt Iodine Level and Associated factors in dire Dawa City Administration, Eastern Ethiopia. East Afr J Health Biomedical Sci.

[44] Bazezew MM, Yallew WW (2018). Belew Ak. Knowledge and practice of iodized salt utilization among reproductive women in Addis Ababa City. BMC Res Notes.

[45] Jooste PL, Weight MJ, Lombard CJ (2001). Iodine concentration in household salt in South Africa. Bull World Health Organ.

[46] Kebebew E, Derese T, Bogale G, Berhane A (2023). Iodine concentration level, availability of adequately iodised salt and proper utilisation, and its influencing factors among households in Eastern Ethiopia: a community-based cross-sectional study. BMJ Open.

[47] Achille LY, MizÃ C, Agbokponto E, Amoussou A (2022). Salt production and availability of Iodized Salt in the municipality of SÃ¨ mÃ¨-Kpodji in 2022. Nutr Food Sci Int J.

[48] Habib MA, Alam MR, Ghosh S, Rahman T, Reza S, Mamun S (2021). Impact of knowledge, attitude, and practice on iodized salt consumption at the household level in selected coastal regions of Bangladesh. Heliyon.

[49] Yeshaw Y, Alem AZ, Tesema GA, Teshale AB, Liyew AM, Tesema AK (2020). Spatial distribution and determinants of household iodized salt utilization in Ethiopia: a spatial and multilevel analysis of ethiopian demographic and Health survey. BMC Public Health.

