# Supplementary material for: Nearly one-in-five households utilized inadequate iodized salt in Nifas Silk Sub-City, Addis Ababa, Ethiopia
Source: BMC Nutr. 2023 Aug 7;9:96. doi: 10.1186/s40795-023-00754-5 (PMC10405470; doi:10.1186/s40795-023-00754-5)
Supplement: Supplementary file 1 — Supplementary Material 1 [file 40795_2023_754_MOESM1_ESM.doc]

**ANNEX-II Questionnaire**

**Jimma University**

**Department human nutrition and dietetics**

**Iodized salt utilization and associated factors among household in woreda 11, Nifas Silk sub city, Addis Ababa, Ethiopia 2020**

| Q.No | Question | Choices | | Skip | | Remark | |
| --- | --- | --- | --- | --- | --- | --- | --- |
| **Part One: Socio demographic and economic characteristics of the respondent.** | | | | | | | |
| 101 | Sex of respondents | | ….. | |  | |  |
| 102 | How old are you?(age in years) | | ………………….. | |  | |  |
| 103 | What is your current marital status? | | 1. Married  2. Single  3. Divorced  4. widowed | |  | |  |
| 104 | Educational status | | 1.Unable to read and write  2.Read and write only  3.Primary school (Grade 1–8)  4.Secondary school(Grade 9-10  5.Preparatory school(Grade 11-12)  6.College and above | |  | |  |
| 105 | Occupation | | 1. Government employ. 2. Merchant Maid 3. Student 4. House wife 5. Bartender 6. Others | |  | |  |
| 106 | Family Size | | ----------------------------- | |  | |  |
| 107 | What is your monthly House hold income (in average) in birr? | | ………. | |  | |  |

| **Part 2 Knowledge of household about iodized salt** | | | | | | | | | | |
| --- | --- | --- | --- | --- | --- | --- | --- | --- | --- | --- |
| 201 | Knows effects of iodine deficiency At least one | 1. Yes 2. No | | | | | |  | |  |
| 202 | Heard about iodized salt | 1. Yes 2. No | | | | | |  | |  |
| 203 | Knows effects of iodine deficiency Two or more | 1. Yes 2. No | | | | | |  | |  |
| 204 | Knows that iodized salt prevents IDD | 1. Yes 2. No | | | | | |  | |  |
| 205 | Source of information on utilizing iodized salt | 1. From social media 2. Health professional 3. Other | | | | | |  | |  |
| **Part 3 Attitude in handling iodized salt at household level** | | | | | | | | | | |
|  | Likers scale | Strongly agree | Agree | Uncertain | Disagree | | S. Disagree |  | |  |
| 301 | I believe that food prepared with iodized salt tastes less delicious than non-iodized salt does |  |  |  |  | |  |  | |  |
| 302 | I believe that iodized salt incurs more cost than its benefits |  |  |  |  | |  |  | |  |
| 303 | I believe that giving iodized salt to small children in advisable |  |  |  |  | |  |  | |  |
| 304 | I believe that iodized salt should be handled in the store, household etc with great care than non-iodized salt |  |  |  |  | |  |  | |  |
| 305 | I believe in advising my family - members to use iodized salt |  |  |  |  | |  |  | |  |
| 306 | I believe in using iodized salt than non-iodized salt |  |  |  |  | |  |  | |  |
| **Part 4Practices of in handling iodized salt at household level** | | | | | | | | | | |
| 401 | Type of salt | - 1. Packed   2. Not packed | | | |  | | |  | |
| 402 | Expose salt to sun light | 1. Yes 2. No | | | |  | | |  | |
| 403 | Place of salt storage | 1. Dry place 2. Moisture area | | | |  | | |  | |
| 404 | Type of container | 1. Container with a lid 2. Container without a lid | | | |  | | |  | |
| 405 | When did they add salt while cooking?? | 1. While cooking 2. After cooking | | | |  | | |  | |
| **Part 5 Availability, Accessibility and Taste of Iodized salt** | | | | | | | | | | |
| 501 | Do you easily get your choice of salt when you need it? | 1. Yes 2. No | | | |  | | |  | |
| 502 | How would you consider the price of your choice of salt? | 1. Expensive 2. Affordable | | | |  | | |  | |
| 503 | Does an iodized salt taste differently from salt with no chemical (iodine) added? | - - 1. Yes     2. No | | | |  | | |  | |
| **Part 6Iodine measurement format? (**filled by the investigator) | | | | | | | | | | |
| 601 | Concentration of iodine in the salt collected from the households | 1. ≥ 15 ppm iodine 2. <15 ppm iodine 3. 0 ppm iodine | | | |  | | |  | |
